# A Case Report of Subcutaneously Injected Liposomal Cannabidiol Formulation Used as a Compassion Therapy for Pain Management in a Dog

**DOI:** 10.3389/fvets.2022.892306

**Published:** 2022-04-28

**Authors:** Yael Shilo-Benjamini, Ahuva Cern, Daniel Zilbersheid, Atara Hod, Eran Lavy, Dinorah Barasch, Yechezkel Barenholz

**Affiliations:** ^1^Laboratory of Membrane and Liposome Research, Department of Biochemistry, Hadassah Medical School, The Hebrew University of Jerusalem, Jerusalem, Israel; ^2^Koret School of Veterinary Medicine, The Robert H. Smith Faculty of Agriculture, Food and Environment, The Hebrew University of Jerusalem, Rehovot, Israel; ^3^The Mass Spectrometry Unit, School of Pharmacy, The Hebrew University of Jerusalem, Jerusalem, Israel

**Keywords:** analgesia, cannabidiol, CBD, dog, liposomes, pharmacokinetics, prolonged release

## Abstract

A 14-year-old intact mixed breed dog (26 kg) was submitted for a novel cannabidiol (CBD) analgesic treatment. The dog was cachectic and had a testicular neoplasia, hip and elbow osteoarthritis and severe cervical pain. Analgesic treatment included canine osteoarthritic supplement, robencoxib and gabapentin. An additional liposomal CBD injectable formulation at 5 mg/kg was administered subcutaneously between the shoulder blades. The dog was monitored using an activity monitoring collar (PetPace), owner wellbeing questionnaire (Canine Brief Pain Inventory; CBPI), pain interactive visual analog scale (iVAS), blood work and CBD plasma concentrations. A week from the injection and up to 3 weeks afterwards the dog had improved CBPI and iVAS pain scores, and increased collar activity scores. CBD was quantified in plasma for 28 days. Due to disease progression, further difficulty to rise and walk, and relapse to pain after 3 weeks, the owners requested a second liposomal CBD injection, which was performed 4 weeks following the first injection using 3 mg/kg dose. Two days later, the dog was found dead in the yard under direct sun, while environmental temperature was 37°C. Major findings on necropsy revealed evidence of heat stroke and severe cervical disc protrusion with spinal hematoma, none related to liposomal CBD. In conclusion, subcutaneous liposomal CBD produced quantifiable CBD plasma concentrations for 28 days and may be an effective additional treatment as part of multimodal pain management in dogs.

## Introduction

Various diseases, such as osteoarthritis, intervertebral disc disease and cancer are common in companion animals and usually result in chronic pain that affects animals' wellbeing (1, 2). Non-steroidal anti-inflammatory drugs (NSAIDs) are commonly used for analgesia of chronic pain in dogs (3–5) but may fail to control pain and can be associated with adverse effects (6, 7).

Cannabinoids are a family of chemicals that act on the endocannabinoid system. Studies of the cannabinoid receptors have shown that endocannabinoids attenuate and suppress the perception of pain (8, 9). Naturally, cannabinoids are produced from cannabis plants, with more than 100 cannabinoids identified (10). The main cannabinoids produced are tetrahydrocannabinol (THC) and cannabidiol (CBD), but in contrast to the highly psychoactive THC, CBD has virtually no psychoactive properties (10, 11). In people, there is evidence that CBD may be a useful treatment for various chronic pain conditions (11–13). The effectiveness of CBD via oral or oral-transmucosal administration for the treatment of osteoarthritis in dogs has also been recently reported (14–17). A safety and pharmacokinetic evaluation of repeated oral CBD administration in healthy dogs showed that oral daily administration of up to 12 mg/kg/day for 28 days was well tolerated, with no clinically important adverse effects (18). Oral CBD administration requires frequent dosing (twice a day) (14, 16), and may result in variable plasma levels due to low bioavailability, estimated in people to be only 6% (19). In dogs, bioavailability of new oral preparations is unknown, as no recent study compared oral to intravenous administration. However, mean CBD plasma levels following single administration were reported to be 300 ng/mL (20) and at steady-state in the range of 60-300 ng/mL (17, 21, 22) depending on dose and oral formulation.

Palatability of oral oil-based CBD preparations is also a downside of oral treatment (23). In an effort to increase CBD bioavailability and allow a more convenient use with a better owner and pet compliance, alternative delivery routes of CBD (i.e., injectable) are of interest. Liposomes are closed vesicles made of one or more bilayers of well-characterized phospholipids. They are attractive for pharmaceutical application because they are biocompatible, biodegradable, non-toxic, their bio-fate is known, and they are already proven as successful drug delivery systems (24–26), including US Food and Drug Administration (FDA) approved liposomal drug-products (26). Encapsulation of CBD into liposomes can facilitate sustained and controlled drug release and may provide long-term significant CBD plasma concentrations that will enable analgesia.

The purpose of this case report is to describe the addition of a novel analgesic treatment (i.e., on top of commonly used analgesics) using liposomal CBD formulation administered subcutaneously as a compassion therapy in a dog suffering from severe chronic pain.

## Case Description

### Clinical History

A 14-year-old intact mixed breed dog with a body weight of 26 kg was referred to Koret School of Veterinary Medicine—Teaching Hospital for a novel analgesic treatment. The dog was cachectic (BSc 3/9) with general muscle atrophy. He suffered from bilateral hip and elbow osteoarthritis (hip confirmed radiographically) and severe cervical pain. The owners preferred not to perform diagnostic tests for the cervical disease. On physical examination the dog was lying down in a lateral recumbency, and required the owner's help to get up due to extreme neck pain. He had proprioception deficits in all 4 limbs, which was worse on the right thoracic limb, and had severe lameness in all limbs. Additionally, the dog had a suspected testicular neoplasia (asymmetric testicles; the enlarged testicle was approximately 6 times larger than the second testicle). The owners reported that the testicular mass doubled its size in the past 6–8 months.

The dog was treated with a canine osteoarthritic supplement twice daily (Super-Flex GLM, Deer Velvet, Dogs 750 mg, SuperFlex, Israel; started 10 months before intervention), and with oral analgesics, including robencoxib 40 mg once daily (Onsior, Elanco, USA; started 6 months before intervention) and gabapentin 200 mg twice daily (Gabapentin, VetMarket, Israel; started 2.5 weeks before intervention).

The owners reported that during the past month the dog deteriorated rapidly, his pain increased, and his function decreased. He stopped eating commercial dry dog food and was eating mostly food prepared from the kitchen of the owners, primarily consisting of meat and chicken products and some canned dog food. The owners were reluctant to euthanize the dog at that time and requested to proceed with the novel CBD treatment.

### Liposomal CBD Intervention

Liposomal CBD formulation was obtained from Innocan Pharma (Israel). According to the product certificate of analysis, the Liposomal CBD was prepared under strict aseptic conditions. Prior to use, samples were sent for tests of sterility and endotoxin levels performed by Hy-Labs (Rehovot, Israel), which is a certified and accredited laboratory by the Israeli Ministry of Health and by the FDA. The results of these tests meet the requirements of extra-vascular administered drugs in humans.

The liposomal CBD formulation was composed of hydrogenated soy phosphatidylcholine (HSPC; Lipoid GmbH, Ludwigshafen, Germany). A synthetic CBD (Purisys LLC., Athens, GA, USA; not considered a controlled substance) was loaded into the liposomes. The external medium was 5% dextrose solution containing 0.075% histidine at pH 7.0. Liposomal CBD characteristics: (i) gross appearance “milky” liquid (ii) median particle size 5.6 μm; (iii) osmolality 317 mOsm/kg; (iv) total CBD concentration 50 mg/g; (v) HSPC concentration 49 mg/g, drug to lipid molar ratio 2.5; (vi) sterility conforms; (vii) pyrogen test <5 EU/mL.

The hair between the shoulder blades of the dog was clipped and the skin was scrubbed with chlorhexidine and then cleaned with alcohol 70%. Liposomal CBD (batch: Lipo-CBD F1, Inn4-117, preparation date 15 June 2021; 50 mg/mL) 2.6 mL (i.e., 5 mg/kg; a total of 130 mg) was aspirated using 18-gauge, 1.5-inch needle from the vial using aseptic techniques and injected subcutaneously with a 21-gauge, 1-inch needle at the prepared skin area.

### Monitoring and Follow Up

**Figure 1:**
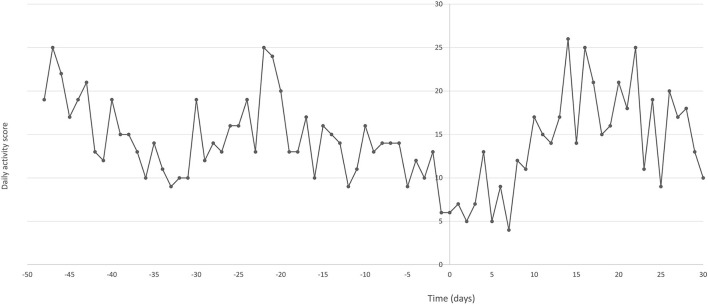
Daily average activity scores collected via an activity monitoring collar (PetPace) from a 14-year-old dog suffering from bilateral hip and elbow osteoarthritis and severe neck pain, before and after liposomal cannabidiol (CBD) subcutaneous injection. X-axis represents days before (negative) and after (positive) liposomal CBD injection on day 0.

Owner questionnaire using the Canine Brief Pain Inventory (CBPI) (27, 28) was completed at baseline, on the day of liposomal CBD intervention, and then once weekly up to 4 weeks (Table 1). Interactive Visual Analog Scale (iVAS) was performed by a board-certified anesthesiologist at the same time points (Table 1). An activity monitoring collar (PetPace, Burlington, MA, USA; https://petpace.com/smart-sensing-collar/) was placed on the dog 7 weeks before intervention via the referring veterinarian. Daily average activity data collected by the collar is presented in Figure 1.

**Table 1:** Scoring of Canine Brief Pain Inventory (CBPI; scale total pain 0–40, scale total function 0–60) by owners and interactive Visual Analog Scale (iVAS; scale 0–10) by an anesthesiologist in a 14-year-old dog suffering from bilateral hip and elbow osteoarthritis and severe neck pain, before and after liposomal cannabidiol subcutaneous injection.

	**Baseline**	**Week 1**	**Week 2**	**Week 3**	**Week 4**
**Description of Pain**					
Worst	8	9	8	7	8
Least	0	0	0	0	0
Average	5	4	4	3	5
Right now	8	4	4	0	1
***Total Pain***	**21**	**17**	**16**	**10**	**14**
**Description of function**					
General activity	9	9	9	9	9
Enjoyment of life	9	8	8	9	9
Ability to rise	9	8	8	8	8
Ability to walk	9	8	8	8	8
Ability to run	10	10	10	10	10
Ability to climb	10	10	10	10	10
***Total function***	**56**	**53**	**53**	**54**	**54**
**Overall**	Poor	Poor	Poor	Poor	Poor
**iVAS**	**10**	**8**	**7**	**7**	**8**

**Figure 2:**
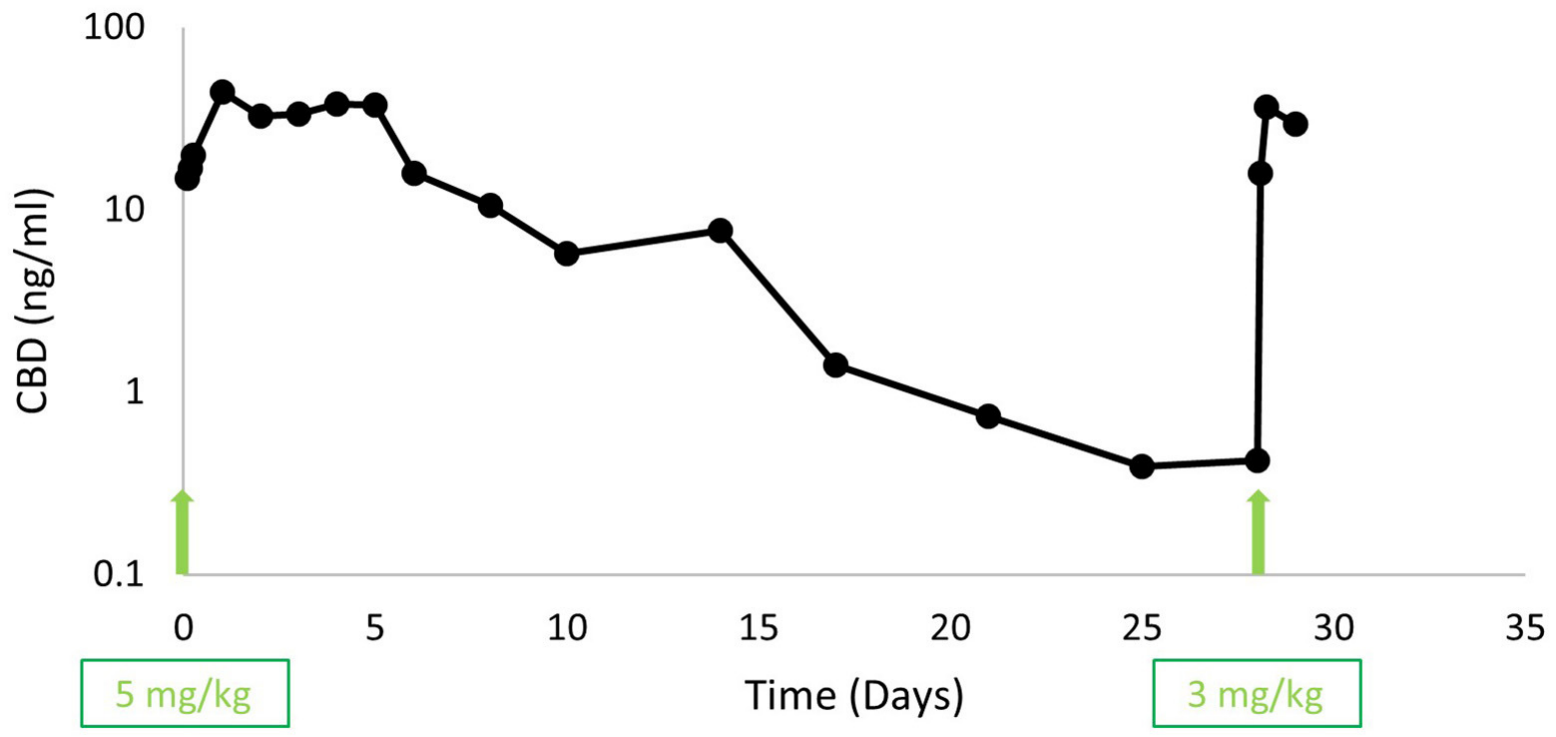
Plasma cannabidiol (CBD) concentrations (ng/mL) in a 14-year-old dog suffering from bilateral hip and elbow osteoarthritis and severe neck pain. The arrows indicate subcutaneous liposomal CBD injections that were performed on days 0 (5 mg/kg) and 28 (3 mg/kg).

Complete blood count (CBC) and biochemistry panel were performed 10 days prior to intervention and then 3 days, 2 weeks, and 4 weeks following liposomal CBD injection (Table 2). The major finding in the CBC was anemia before injection, which was worse after intervention and may have been attributed to the frequent blood collection combined with the apparent decreased appetite immediately after injection. White blood cells were increased on day 3 after injection, and by 2 weeks returned to baseline values. Blood (1-2 mL) was also collected for pharmacokinetic analysis at 2, 4, and 6 h, daily at 1–6 days, 8-, 10-, 14-, 17-, 21-, 25-, and 28-days following injection (Figure 2). Blood was collected into ethylenediamine tetra-acetic acid (EDTA) tubes, kept in ice, and centrifuged to separate the plasma within 20 min. Plasma was immediately frozen at −20°C and then kept at −80°C until analysis. CBD quantification was performed using UHPLC-tandem mass spectrometry (LC-MS/MS) method (Supplementary Material). Pharmacokinetic parameters were calculated for 28 days following injection using a non-compartmental analysis using Phoenix WinNonlin (CertaraTM, NJ, USA, Version 6.3).

**Table 2:** Complete blood count and biochemistry panel performed in a 14-year-old dog suffering from bilateral hip and elbow osteoarthritis and severe neck pain, before and after liposomal cannabidiol subcutaneous injection.

**Parameter**	**Reference range**	**Baseline-10 days**	**3 days**	**2 weeks**	**4 weeks**
**Hematology**
White blood cells (10^9^/L)	5.0–22.0	8.98	14.32	8.18	5.88
Neutrophils (10^9^/L)	2.0–12.8	7.52	12.29	6.41	5.05
Lymphocytes (10^9^/L)	0.6–12.8	0.89	1.16	0.83	0.49 L
Monocytes (10^9^/L)	0.0–2.8	0.52	0.84	0.83	0.33
Eosinophils (10^9^/L)	0.0–2.0	0.04	0.03	0.07	0.01
Basophils (10^9^/L)	0.0–0.5	0.00	0.01	0.03	0.00
Neutrophils (%)	50.0–80.0	83.76 H	85.79 H	78.41	85.96 H
Lymphocytes (%)	12.0–30.0	9.91 L	8.11 L	10.20 L	8.31 L
Monocytes (%)	0.0–14.0	5.83	5.85	10.14	5.55
Eosinophils (%)	0.0–10.0	0.45	0.20	0.90	0.14
Basophils (%)	0.0–2.5	0.05	0.04	0.36	0.04
Hematocrit (%)	35.8–60.0	31.6 L	27.6 L	26.8 L	34.2 L
Red blood cells (10^12^/L)	4.35–9.20	4.34 L	3.50 L	3.67 L	4.65
Hemoglobin (g/dL)	11.0–19.5	10.1 L	8.1 L	9.6 L	10.8 L
Mean corpuscular volume (fL)	60.0–80.0	72.7	79.0	73.1	73.6
Mean corpuscular hemoglobin (pg)	19.0–24.5	23.3	23.1	26.2 H	23.2
Mean corpuscular hemoglobin concentration (g/dL)	30.0–36.0	32.0	29.3 L	35.8	31.6
Red cell distribution width (%)	10.5–18.0	13.2	16.4	13.7	13.3
Reticulocytes (10^9^/L)	0.0–60.0	59.5	140.4 H	40.4	39.1
Reticulocytes (%)	0.0–1.2	1.37 H	4.01 H	1.10	0.84
Platelets (10^9^/L)	200–500	351	518 H	427	467
Plateletcrit (%)		0.435	0.611	0.474	0.542
Mean platelet volume (fL)	6.0–17.0	12.4	11.8	11.1	11.6
Platelets distribution width (%)		24.9	25.1	26.5	25.1
Packed Cell Volume (%)		31	25	30	33
Total solids		6.4	7.0	6.4	6.4
**Biochemistry**
Albumin (g/dL)	2.5–4.4	3.3	3.2	3.1	3.2
Alkaline phosphatase (U/L)	20–150	47	66	54	50
Alanine transaminase (U/L)	10–118	24	22	29	43
Amylase (U/L)	200–1,200	559	714	493	469
Total bilirubin (mg/dL)	0.1–0.6	0.3	0.4	0.3	0.3
Blood urea nitrogen (mg/dL)	7–25	11	17	12	11
Calcium (mg/dL)	8.6–11.8	9.3	9.3	9.1	9.1
Phosphorus (mg/dL)	2.9–6.6	3.0	4.3	5.5	5.7
Creatinine (mg/dL)	0.3–1.4	0.7	0.9	0.6	0.8
Glucose (mg/dL)	60–110	127	93	97	107
Sodium (mmol/dL)	138–160	145	147	146	146
Potassium (mmol/dL)	3.7–5.8	3.4	4.1	4.7	4.2
Total protein (g/dL)	5.4–8.2	6.2	6.8	6.4	6.6
Globulins (g/dL)	2.3–5.2	2.8	3.6	3.3	3.3

*High (H) or low (L) relative to the “Reference range” values column for healthy dogs*.

The car ride to the Hospital and back on the day of intervention seemed to worsen the dog's pain and function. On the first several days following Hospital visit, the dog required the owner's help to get up, had significant difficulty while walking and had a selective eating and drinking behavior. Therefore, the dog was administered subcutaneous 500 mL of lactated ringer's solution twice a day for 3 days. Thereafter he recovered gradually, and had improved owner and iVAS pain scores, and increased collar activity scores (compared to his activity before the injection) up to 3 weeks following injection (Table 1, Figure 1). The improvement in the pain score correlated with the pharmacokinetic profile obtained (Figure 2). CBD plasma levels increased gradually during the first 24 h after injection to a maximum concentration of 44 ng/mL and remained in the range of 33–38 ng/mL for the next 4 days. The concentrations decreased in a mono exponential decline thereafter with CBD plasma concentration of 0.4 ng/mL at day 28 after injection. The AUC obtained over the 28 days was 6,966 ng·h/mL and half-life was 86 h.

Approximately 3 weeks from injection, the dog showed signs of cervical disease progression, further difficulty to use its right thoracic limb, to rise and walk, and it relapsed to severe pain. Therefore, the owners requested a second liposomal CBD injection, which was performed 4 weeks following the first injection using a lower dose of 3 mg/kg. The injection was performed at the owners' home (in order to avoid the car-ride deterioration) with direct veterinary supervision for 7 h following injection. Blood samples were collected for pharmacokinetic analysis at 2 and 6 h, and 1 day following injection (Figure 2). Two days later, the dog was alone in the yard for several hours, while environmental temperature was high (37°C). When the owners went outside, they found the dog deceased under direct sun. Major findings on necropsy revealed evidence of heat stroke (diffuse cerebral and meningeal edema and congestion, several focal cerebral hemorrhages, diffuse alveolar edema and congestion including thick mucoid material in the bronchi and foam in the trachea, diffuse muscular edema with multifocal muscular hemorrhage and focal subcapsular renal and hepatic hemorrhage). He had a severe cervical disc protrusion with a large blood clot in the intervertebral canal at the level of cervical vertebrae 1–4. Additional findings included severe bilateral femoral head cartilage degeneration, testicular seminoma, benign prostatic hyperplasia, bladder cyst, large hematoma at the back muscles on the left side at the level of thoracic vertebrae 10–13, moderate hepatic lipidosis, an acute left kidney infarct and bilateral adrenal hyperplastic noduli. The dog was eating and drinking as usual during the 2 days after the second injection, including several hours before his death, and the cause of death was suspected to be heat stroke. It was assumed that the dog fell due to his condition or due to a brain stroke (observed focal cerebral hemorrhages), was not able to get up, and because he was lying in the sun, he developed heat stroke. This assumption was also supported by data downloaded later from the monitoring collar, which showed increased temperature (from “Normal” to “High”), increased pulse rate (from 70 to over 200 beats per minute) and decreased heart rate variability (HRV; from 11.5 to 8.0) during two h before the suspected time of death. Unfortunately, during the event, because the dog was outside and too far away from the router, data was not transferred in real-time and collar alerts were not sent to the owner and the veterinarian.

Although no local changes were grossly observed in response to injection, skin and subcutaneous tissues from the two liposomal CBD administration sites were also sampled, fixed in formaldehyde 4% and assessed histologically. The first area injected (30 days following injection) presented normal tissue with no microscopic changes identified. The second area injected (2 days following injection) presented a cavity that contained wisps of fibrin-like material and a small number of neutrophils. Multifocal congestion and edema were observed, and near the cavity edge a moderate neutrophil infiltration of the adipose tissue was identified. Histopathologic findings were compatible with fibrinopurulent cellulitis.

## Discussion

In this report, subcutaneous liposomal CBD administration provided long-term CBD plasma concentration, although lower than what has been reported during steady-state following repeated daily oral administration (22, 23), and it seemed to improve dog's pain and owner perceived function up to 3 weeks from injection. CBD was reported to provide analgesia in people (9, 12) and recently in dogs with osteoarthritis (14–17). The mechanism of action suggested for CBD analgesic effect is mediated via cannabinoid receptor 2 as an inverse agonist and as an inhibitor of the reuptake of the endocannabinoid anandamide (13, 29). Additionally, CBD was reported to work on various other receptors, including the serotonin receptor 5-HT_1A_ and the transient receptor potential cation channel, subfamily vanilloid 1 receptor (TRPV1). TRPV1 channels are predominantly expressed in sensory neurons and have an important role in nociception (13, 30). Regarding joint pain, an anti-nociceptive effect of locally administered CBD has been reported, attributed to decreased joint nociceptors firing and decreased local inflammation, which resulted in decreased pain behavior in an end-stage osteoarthritis model in rats (11, 31).

Osteoarthritis impact on chronic pain and activity levels in dogs has been documented and it has been suggested that improvement in mobility could be one of the therapeutic goals (32). In this report the dog had an activity monitor, which provided a more objective measurement of activity (33). PetPace is a non-invasive collar designed for dogs and cats that allows real-time, continuous, remote monitoring of activity, temperature, pulse, respiration, variations in heart rate (HRV), position (lying, sitting, standing), and sleep quality (movement during sleep). The use of this collar was recently described in dogs and showed an excellent correlation with real-time variables (33–35). The results of increased activity following the liposomal CBD treatment in the dog presented here seemed to be correlated with owner subjective wellbeing evaluation and iVAS, although, this data should be interpreted with caution as other factors such as environmental conditions and owner activity can play a significant role in daily activity patterns.

Changes in blood work were observed on day 3 following injection. There was a decrease in hematocrit, which was already low at baseline and was attributed to the frequent blood sampling on the first days. White blood cells (WBCs) were increased, but were not above the reference range, and were back to baseline 10 days later. The increase in WBCs can be explained by the immune system response to large size particles (36, 37) and can be correlated to the local fibrinopurulent cellulitis observed on histopathology 2 days following the second injection. This observation of local response followed by recovery was also reported in other studies investigating other liposomal formulations (38) or administration of other particle vehicles (36). Oral CBD administration for several weeks in dogs resulted in increased serum alkaline phosphatase (ALP), likely due to induction of CYP isoenzymes in the liver (20, 23). ALP change was not observed in the present report, possibly because injectable route bypasses hepatic metabolism.

Liposome-based formulations provide sustained-release of drugs (24, 26), and in many cases improve drug therapeutic index (39). In this report, a single subcutaneous injection of CBD encapsulated into liposomes was proven to provide slow drug release over the tested period. The reported oral bioavailability of CBD is 6% in people and considered to be variable and dependent on fasting/fed conditions (19). Parenteral CBD administration, such as reported here, is beneficial as it is not dependent on food intake and absorption may be complete avoiding the first pass metabolism. The AUC obtained in the present report was 6,966 ng·h/mL and when normalized to dose it is 1,393 ng·h/mL/mg/kg. This value is similar to the AUC obtained following CBD intravenous administration to dogs, which was 1,203 and 1,354 ng·h/mL/mg/kg following mean doses of 2.25 and 4.5 mg/kg (45 mg and 90 mg to dogs weighing 16–24 kg), respectively (40). In that study, an oral CBD in gelatin formulation using 180 mg per dog (i.e., a mean dose of 6 mg/kg) resulted in bioavailability of 13–19% in 3 of the 6 dogs tested and 0% (plasma CBD was not detected) in the other 3 dogs. If bioavailability is calculated for the dog presented here (AUC subcutaneous / Dose [mg/kg] divided by AUC intravenous / Dose [mg/kg]), then the bioavailability obtained after subcutaneous administration of the described liposomal CBD is complete (i.e., 1,393 ng·h/mL/mg/kg / 1,354 ng·h /mL/mg/kg = 1.03). While intravenous administration outlined high initial plasma concentrations and rapid decline of CBD plasma levels followed by a prolonged elimination, with a mean half-life of 7–9 h, the CBD plasma profile obtained with subcutaneous liposomal administration increased gradually and showed mono exponential decline starting 6 days following administration, with half-life of 86 h.

Pharmacokinetic evaluation of repeated oral CBD administration 1–12 mg/kg/day for 28 days in healthy dogs reported an AUC normalized to dose of 119 ng·h/mL/mg/kg after the 1st dosing day and 241 ng·h/ml/mg/kg after the 28th day of the highest dose tested (12 mg/kg) (18). These values represent 9% and 18% bioavailability, respectively, compared with intravenous administration (40). The reported C_max_ and T_max_ at the 28th day of administration were 53–201 ng/mL and 2.3–5.8 h, respectively (18). Another study investigating the pharmacokinetics of a single-dose CBD oil (2 vs. 8 mg/kg) in four beagle dogs reported an AUC normalized to dose of 184 and 332 ng·h/ml/mg/kg, which may correspond to a bioavailability of approximately 14 and 25%, respectively. Median C_max_ and T_max_ were 102.3 ng/mL and 1.5 h and 590.8 ng/mL and 2 h for the 2 and 8 mg/kg, respectively, with half-life of 4.2 h following both doses (14). A study investigating the pharmacokinetics of CBD in an oral formulation of cannabis herbal extract containing 1:20 THC:CBD at three CBD doses: 2, 5 and 10 mg/kg (each dose tested in 6 dogs), reported a normalized to dose AUC of 380, 587 and 724 ng·h/ml/mg/kg, respectively (41). Because the variance in that study was very high, bioavailability could not be determined. C_max_ and T_max_ reported in that study were 213–1,868 ng/mL and 1.9–2.3 h, respectively, with half-life of approximately 2.5 h following all doses (41). It is important to note that in recent studies CBD dosage forms were oil-based, which produced higher CBD plasma concentrations compared with the concentrations reported by Samara et al. that used oral delivery of water soluble gelatin-based preparation (40). The higher plasma CBD is likely related to improved CBD absorption.

In the present report C_max_ was lower than reported steady-state CBD plasma/serum concentration following 2–6 weeks oral administration of CBD or hemp extract in several studies in dogs; approximately 60–125 ng/mL (22), approximately 80–160 ng/mL (21), median (range) 311 (5–860) ng/mL (17). Although higher doses can be administered when prolonged-release formulations are used, in the present report we decided to use a generally moderate dose, which was also tested following intravenous administration (40), because the pharmacokinetic profile of the formulation was unknown in dogs. In further studies of the liposomal CBD, pharmacokinetics and clinical effects of higher doses should be investigated.

Cannabis products for veterinary patients may not be legally permitted in some countries, which limits their beneficial use in painful animals. However, synthetic CBD, as was used in the present report, is not considered a controlled substance, and it may be used in the future in veterinary patients, although, more evidence-based research is indicated.

Limitations to this case report include the fact that this formulation was tested only in one dog and other dogs may respond differently and/or adverse effects may be produced. The dog was geriatric, which may have affected CBD absorption and elimination from the body. Additionally, his old age and poor systemic condition differ from most experimental dogs from other studies, rendering the comparison of his AUC to these studies not meaningful. Bioavailability was calculated based on the only study reporting intravenous CBD administration from 1988 (40), which is suboptimal and probably not accurate. Both owners and veterinarian were aware of the treatment, and it is known that care giver bias may affect patient evaluation.

## Conclusion

Subcutaneous administration of liposomal CBD showed high exposure in terms of AUC, with relatively lower CBD plasma concentrations maintained over the tested period (28 days) and may be an effective and attractive additional treatment as part of multimodal pain management in dogs suffering from chronic pain. This formulation can be an alternative route to oral CBD administration in cases where owner compliance, palatability and/or bioavailability are low. Further investigations of this formulation are of interest.

## Data Availability Statement

The original contributions presented in the study are included in the article/Supplementary Material, further inquiries can be directed to the corresponding author.

## Ethics Statement

Ethical review and approval was not required for the animal study because this case report was carried out as a compassion treatment in accordance with “Good Clinical Practice”. Written informed consent was obtained from the owners for the participation of their animals in this study.

## Author Contributions

YS-B, AC, and DB contributed to the data acquisition and interpretation, drafted, and revised the manuscript. DZ and AH contributed to the data acquisition. YB and EL interpreted the results and revised the manuscript. All authors approved the final version to be submitted.

## Funding

The liposomal CBD formulation and all treatments and tests were funded by Innocan Pharma Ltd., Israel.

## Conflict of Interest

AC, DZ, and AH are supported by Innocan Pharma Ltd. AC and YB have a patent pending on the liposomal CBD used in this report. The remaining authors declare that the research was conducted in the absence of any commercial or financial relationships that could be construed as a potential conflict of interest.

## Publisher's Note

All claims expressed in this article are solely those of the authors and do not necessarily represent those of their affiliated organizations, or those of the publisher, the editors and the reviewers. Any product that may be evaluated in this article, or claim that may be made by its manufacturer, is not guaranteed or endorsed by the publisher.
