# Supplementary material for: A Case Report of Subcutaneously Injected Liposomal Cannabidiol Formulation Used as a Compassion Therapy for Pain Management in a Dog
Source: Front Vet Sci. 2022 Apr 28;9:892306. doi: 10.3389/fvets.2022.892306 (PMC9097221; doi:10.3389/fvets.2022.892306)
Supplement: Supplementary file 1 [file Data_Sheet_1.docx]

**Supplementary Material**

**Determination** **of CBD in plasma by LC-MS/MS**

*LC-MS/MS instrument*

LC-MS/MS analyses were conducted on a Sciex (Framingham, MA, USA) Triple Quad™ 5500 mass spectrometer coupled with a Shimadzu (Kyoto, Japan) UHPLC System.

*UHPLC conditions*

The chromatographic separations were performed on a CORTECS® (Waters Corp., Milford, MA, USA) column (C18, 2.7 µm particle size, 50 x 2.1 mm), protected by a VanGuard® (Waters Corp., Milford, MA, USA) pre-column. The injection volume was 5 μL, the oven temperature was maintained at 40°C and the autosampler tray temperature was maintained at 5°C.

The chromatographic separation was achieved using a linear gradient program at a constant flow rate of 0.3 mL/min over a total run time of 15 min. An outline of the mobile phase gradient program is summarized in **Table 1S**.

**Table 1S**: Gradient program: Solvent A is 0.1% FA in water and solvent B is ACN.

| Time (min) | Solvent A (%) | Solvent B (%) |
| --- | --- | --- |
| 0.0 | 75 | 25 |
| 0.5 | 75 | 25 |
| 1.5 | 58 | 42 |
| 6.0 | 45 | 55 |
| 8.5 | 20 | 80 |
| 10.0 | 2 | 98 |
| 12.0 | 2 | 98 |
| 12.5 | 75 | 25 |
| 15.0 | 75 | 25 |

The column effluent was diverted away from the MS during the first 1 min and last 2.0 min of the run. Methanol was used for washing the needle prior to each injection cycle. All samples were analyzed in duplicate.

*MS/MS conditions*

CBD and the internal standard (IS) cannabigerol (CBG) were detected in positive ion mode using electron spray ionization (ESI) and multiple reaction monitoring (MRM) mode of acquisition.

The molecular ions of the compounds [M+H]^+^ were selected in the first mass analyzer and fragmented in the collision cell followed by detection of the products of fragmentation in the second analyzer. Their transitions are shown in Table 2S. The TurboIonspray^®^ probe temperature was set at 500°C with the ion spray voltage at 4500 V. The curtain gas was set at 25.0 psi. The nebulizer gas (Gas 1) was set to 50 psi, the turbo heater gas (Gas 2) was set to 60 psi and the collision gas (CAD) was set to 8 psi. The entrance potential (EP) was set at 10 V. The collision energy potentials (CE), collision cell exit potentials (CXP) and declustering potentials (DP) for the monitored transitions are given in [Table](http://www.sciencedirect.com/science/article/pii/S0379073811003951#tbl0005) 2S. The dwell time was 30 ms. Data acquisition was performed using Analyst 1.6.3 software and data was analyzed using MutiQuant 2.1 software, both distributed by Sciex. Quantitative calibration (0–400 ng/mL) of CBD was performed before every batch of samples using peak-area ratios (compound versus internal standard). The calibration curve (*y* = *a* + *bx*) was obtained by weighted (1/y) linear least-squares regression of the measured peak-area ratio (*y*) versus the concentration added to the plasma (*x*). The limit of quantification (LOQ) was 0.5 ng/mL for CBD.

**Table 2S**: Multiple reaction monitoring (MRM) transitions and parameters for CBD and CBG (IS) in positive ion mode. m/z: mass to charge ratio; DP: declustering potential; CE: collision energy; CXP: collision cell exit potential; V: volts; eV: electronvolts; Rt: retention time.

| Name | Precursor (*m*/*z*) | Product  (*m*/*z*) | | DP (V) | CE (eV) | | | CXP (V) | Rt (min) | |
| --- | --- | --- | --- | --- | --- | --- | --- | --- | --- | --- |
| CBD | 315.1 | Quantifier | 193.0 | 40 | | 34 | 20 | | | 7.6 |
|  |  | Qualifier | 123.1 | 40 | | 43 | 18 | | |  |
| CBG | 317.1 | Quantifier | 193.1 | 80 | | 23 | 24 | | | 7.7 |
|  |  | Qualifier | 123.1 | 80 | | 43 | 14 | | |  |

*Plasma samples preparation*

CBD was extracted from plasma samples that were spiked with cannabigerol (CBG, 1 mg/ml in methanol, Sigma, Cat. C-141-1) used as internal standard (IS) followed by dilution of the plasma five-fold in acetonitrile. After vigorous vortex it was centrifuged, and the upper phase was analyzed. Final IS concentration in the samples was 100 ng/ml. CBD concentrations were calculated based on a calibration curve of CBD in plasma having 100 ng/ml of IS. In case of low CBD concentrations, the upper phase after dilution in acetonitrile was evaporated to dryness and reconstituted with 100 µl of acetonitrile. In this case, CBD concentrations were calculated from a calibration curve prepared in the same way.
